# Editorial: Regulation of Inflammation in Chronic Disease

**DOI:** 10.3389/fimmu.2019.00737

**Published:** 2019-04-12

**Authors:** Jixin Zhong, Guixiu Shi

**Affiliations:** ^1^Cardiovascular Research Institute, Case Western Reserve University, Cleveland, OH, United States; ^2^Department of Rheumatology and Clinical Immunology, The First Affiliated Hospital of Xiamen University, Xiamen, China

**Keywords:** inflammation, chronic disease, immune regulation, pathogenesis, molecular mechanism

Growing evidence suggests a close link between inflammation and many chronic health conditions including diabetes, metabolic syndrome, cardiovascular disease, cancer, rheumatoid arthritis, inflammatory bowel disease, asthma, and chronic obstructive lung disease. Inflammation is a normal biological defense against infection and tissue damage. Under normal circumstances, it quickly ends after the clearance of infection and injurious agents. There is precise control of the complex networks of inflammatory pathways to limit tissue damage during inflammation. Despite the recognition of the importance of inflammatory dysregulation in chronic illnesses, the mechanisms underlying the inflammatory regulation of these disorders are not fully understood. The current Research Topic issue covers a wide range of subjects in inflammatory regulation in chronic conditions such as obesity (Donninelli et al.; Kim et al.), diabetes (Clark et al.; Purohit et al.), arthritis (Agere et al.; Belmellat et al.; Li et al.; Moon et al.; Pan et al.; Park et al.) cancer (Donninelli et al.; Oghumu et al.), Meniere's disease (Frejo et al.), stroke (Fu and Yan), arteritis (Espígol-Frigolé et al.), asthma (Vroman et al.), erythema nodosum (Negera et al.), systemic lupus erythematosus (Pan et al.), autoimmune encephalomyelitis (Clemente et al.), glomerulonephritis (Hachmo et al.), myasthenia gravis (Wang and Yan), intervertebral disc herniation (Monchaux et al.), inflammatory bowel disease (Liang et al.; Nold-Petry et al.), aging (Ahnstedt et al.), periodontitis (Ren et al.), fibrosis and tissue remodeling (Agere et al.; Ferreira et al.; Li et al.; Paquissi), hepatitis (Ge et al.; Liu et al.; Paquissi), retinopathy (Adamus), and cardiovascular diseases (Katare et al.; Mozos et al.; Qi et al.).

In this Research Topic, Chiurchiù et al. summarized the cellular and molecular mechanisms of endogenous bioactive lipids in the regulation of chronic inflammation. The regulation of NF-κB inflammatory pathway by A20/TNFAIP3 and its involvement in autoimmune diseases was reviewed by Das et al. However, in many chronic conditions, the inflammatory response continues and leads to significant tissue/organ damage and abnormal repair/remodeling (Agere et al.; Ferreira et al. ; Li et al.; Paquissi). Agere et al. demonstrated in their recent work that the CC-chemokine RANTES (regulated on activation normal T-cell expressed and secreted; also called CCL5) is able to activate matrix metallopeptidase-1 (MMP-1) and MMP-13, thus inducing collagen degradation and tissue damage. In contrast, excessive production of extracellular matrix such as collagen would lead to fibrosis and abnormal remodeling, which have also been associated with inflammatory process (Ferreira et al.; Li et al.; Paquissi). Dysregulation of inflammatory response contributes broadly to the development of many chronic conditions. In chronic inflammation, immune cells become dysregulated and loss self-limiting nature. Kim and coworkers identified that macrophage lamin A/C mediates obesity-induced adipose tissue inflammation and insulin resistance by regulating NF-κB. Lamin A/C was increased in the adipose tissue macrophages (ATMs) isolated from obese humans and mice. Lamin A/C overexpression spontaneously activates NF-κB, while lamin A/C deficiency ameliorated obesity-induced insulin resistance and adipose tissue inflammation (Kim et al.). A number of inflammatory cytokines such as TNF-α, IFN-γ, IL-1β, IL-6, IL-17, IL-12, IL-23, and CCL5 are involved in the pathogenesis of chronic disorders (Agere et al. ; Espígol-Frigolé et al.; Negera et al. ; Nold-Petry et al. ; Paquissi; Purohit et al.). Purohit et al. showed that the activity of TNF-α/IL-6 pathway was associated with the risk score of microalbuminuria in patients with type 1 diabetes. Patients with giant-cell arteritis have been shown to have increased levels of IL-12 and IL-23 heterodimers, which could be reduced upon glucocorticoid treatment (Espígol-Frigolé et al.). IL-12/IL-18-induced intestinal inflammation could be alleviated by blockade of heat shock protein gp96 by gp96-II peptide (Nold-Petry et al.). Metabolic reprogramming has been suggested to play an important role during this process. Dumitru et al. reviewed how CD4+ T cells metabolically adapt to different microenvironments during inflammation. Other molecules that regulating the inflammatory pathways discussed in this Research Topic include CCR5 (Vangelista and Vento), TLR (Angelini et al.; Katare et al.), NLRP3 (Li et al.), STAT3/FRA1/JUNB (Moon et al.), TWEAK/Fn14 (Boulamery and Desplat-Jégo; Frejo et al.), glucagon-like peptides (Duan et al.), IL-4 (Tu et al.).

Cortisol and anti-inflammatory natural products are able to provide protections on a number of chronic diseases (Espígol-Frigolé et al.; Lv et al.; Negera et al.; Oghumu et al.; Wang et al.). Espígol-Frigolé et al. reported that patients with giant-cell arteritis had increased levels of IL-12 and IL-23 heterodimers, which were reduced upon glucocorticoid treatment. Negera et al. reported in a case-control study that prednisolone treatment in patients with erythema nodosum leprosum significantly increased the mRNA expression of IL-10 and TGFβ and reduced the expression of TNF, IFN-γ, IL-1β, IL-6, and IL-17A in the blood and skin lesion. Studies have suggested an inverse correlation between increased fruit and vegetable consumption and improved inflammatory conditions (1–3). In this Research Topic, Oghumu et al. showed that dietary black raspberry is able to suppress pro-inflammatory pathways and inhibit oral carcinogenesis. Asiatic acid, a pentacyclic triterpene found in various vegetables and fruits, also exhibited anti-inflammatory and anti-oxidant activities in a fulminant hepatic failure disease model (Lv et al.). Wang et al. also reported that (–)-Epigallocatechin gallate, which is found in Chinese green tea and Pu'er tea, exerts its anti-inflammatory function through inhibiting Notch signaling.

As summarized above, the original research articles and review papers in this issue present a range of topics under active investigation in the area of chronic inflammatory regulation.

## Author Contributions

JZ and GS wrote and approved the manuscript.

### Conflict of Interest Statement

The authors declare that the research was conducted in the absence of any commercial or financial relationships that could be construed as a potential conflict of interest.

## References

[1] Root MM McGinn MC Nieman DC Henson DA Heinz SA Shanely RA. Combined fruit and vegetable intake is correlated with improved inflammatory and oxidant status from a cross-sectional study in a community setting. Nutrients. (2012) 4:29–41. 10.3390/nu4010029 22347616 PMC3277099

[2] Holt EM Steffen LM Moran A Basu S Steinberger J Ross JA. Fruit and vegetable consumption and its relation to markers of inflammation and oxidative stress in adolescents. J Am Diet Assoc. (2009) 109:414–21. 10.1016/j.jada.2008.11.036 19248856 PMC2676354

[3] Hosseini B Berthon BS Saedisomeolia A Starkey MR Collison A Wark PAB. Effects of fruit and vegetable consumption on inflammatory biomarkers and immune cell populations: a systematic literature review and meta-analysis. Am J Clin Nutr. (2018) 108:136–55. 10.1093/ajcn/nqy082 29931038

